# Timing of norepinephrine in septic patients: NOT too little too late

**DOI:** 10.1186/s13054-014-0691-x

**Published:** 2014-12-15

**Authors:** Jean-Sebastien Rachoin, Richard P Dellinger

**Affiliations:** Cooper Medical School of Rowan University, 401 Broadway, Camden, NJ 08103 USA; Division of Hospital Medicine, Cooper University Hospital, One Cooper Plaza, Camden, NJ 08103 USA; Adult Health Institute, Cooper University Hospital, One Cooper Plaza, Camden, NJ 08103 USA; Critical Care Medicine, Cooper University Hospital, One Cooper Plaza, Camden, NJ 08103 USA

## Abstract

After years and years of consensus expert opinion as to mean arterial pressure (MAP) target and vasopressor choice in septic shock management, literature is now emerging that supports the MAP target of 65 mm Hg and norepinephrine as the vasopressor choice. However, the literature remains sparse as to the timing of vasopressors relative to fluid resuscitation and how MAP support is balanced between the choices of vasopressor versus fluid resuscitation. Bai and colleagues report data that reveal an association between earlier vasopressor initiation in septic shock and better outcome. Whether this is a linkage to better care, is related to improved early tissue perfusion, or relates to sparing of fluids to reach the MAP target is not yet known.

## Introduction

In a previous issue of *Critical Care*, Bai and colleagues [1] report the result of a retrospective study of 213 patients with sepsis treated with norepinephrine and analyzed the relationship of the mortality outcome to the timing of initiation of vasopressor. The authors found that patients who had norepinephrine initiated earlier had higher mean arterial pressure (MAP) values, lower lactic acid levels, and lower mortality. This study supports the importance of the role of vasopressors in achieving the MAP target in the early resuscitation of septic shock.

## Initial resuscitation of septic shock

Sepsis is a disease of common occurrence with vexingly high mortality rates despite all of the advances in diagnosis, process changes, and treatment modalities [2,3]. Based on recommendations from the Surviving Sepsis Campaign, if a patient with sepsis is hypotensive within the first 6 hours of diagnosis, the clinician should initiate vasopressor agents to raise the MAP to 65 mm Hg if fluid resuscitation of 30 mL/kg fails to achieve that goal. Not addressed in this study are the optimal choice for vasopressor agent and the optimal target MAP. Other recent studies do address these issues [4-6].

### Timing of initiation of pressor therapy

Bai and colleagues performed a retrospective study of timing of initiation of norepinephrine for treatment of septic shock. They analyzed outcomes of 213 patients admitted to one of two intensive care units. The authors found a strong relationship between delayed initial norepinephrine administration and 28-day mortality.

These findings strengthen the mantra of early initiation of protocolized management of septic shock that includes vasopressor therapy for the early achievement of an MAP target. Perhaps this target should join early diagnosis, early antibiotics, early and appropriate fluid resuscitation, and source control as the essentials of septic shock management [7].

In our mind, there are three potential reasons why earlier initiation of vasopressor therapy is linked to improved outcome. Each one deserves attention as a potential link. Other potential reasons may yet be brought forward.

1. Earlier initiation of vasopressor therapy raises the MAP to a level that facilitates tissue perfusion and prevents onset or progression of organ dysfunction. It is well recognized that organs require a critical MAP to maintain adequate perfusion. When the MAP is allowed to stay below that organ’s critical perfusion pressure, organ injury may occur.
2. Earlier initiation of vasopressor therapy is linked to better knowledge and better management skills for severe sepsis by the treating providers. It may be that earlier vasopressor therapy is a marker of quality of delivered care and this alone would lead to improved outcomes regardless of the impact of the vasopressor alone on outcome.
3. Earlier administration of vasopressor therapy leads to a decrease in the amount of fluids administered over the first 24 hours or more of therapy of septic shock. There is concern that the use of aggressive early fluid resuscitation in septic shock (regardless of whether it is essential for stabilization) leads to potential detrimental increased tissue edema down the road in the patient with septic shock, although this concern is not currently supported by solid evidence. It may be that this edema is associated with organ dysfunction, need for organ support, and the ability to wean organ support. This could lead to longer days on mechanical ventilation, longer days in the intensive care unit, and the potential for later-stage increases in morbidity and mortality. It is possible that aggressive fluid resuscitation saves lives on the front end but a price is paid on the back end. There likely is a fine balance between the use of vasopressors to maintain MAP versus the use of continued fluid resuscitation in the presence of capillary leak to maintain MAP. Much additional research will be required to offer guidance to the treating clinician. In the meantime, one should not err on the side of fluid restriction for the sole purpose of decreasing third spacing down the road. Patients typically autodiurese this fluid when septic shock has resolved and the patient is improving. Whether facilitating this fluid exit with diuretic therapy would be beneficial is currently unknown.

## Conclusions

This trial raises some important questions that need to be answered with large prospective randomized studies: is there an optimal time for initiation of intravenous pressors, and are some of the components of a sepsis protocol more important than others?

## Abbreviation

MAP: mean arterial pressure
